# Molecular Characterisation and Diagnosis of Root-Knot Nematodes (*Meloidogyne* spp.) from Turfgrasses in North Carolina, USA

**DOI:** 10.1371/journal.pone.0143556

**Published:** 2015-11-24

**Authors:** Weimin Ye, Yongsan Zeng, James Kerns

**Affiliations:** 1 Nematode Assay Section, Agronomic Division, North Carolina Department of Agriculture & Consumer Services, Raleigh, North Carolina, United States of America; 2 Department of Plant Pathology, North Carolina State University, Raleigh, North Carolina, United States of America; 3 Department of Plant Protection, Zhongkai University of Agriculture and Engineering, Guangzhou, People’s Republic of China; INRA, FRANCE

## Abstract

Root-knot nematodes (*Meloidogyne* spp.) are the most common and destructive plant-parasitic nematode group worldwide and adversely influence both crop quality and yield. In this study, a total of 51 root-knot nematode populations from turfgrasses were tested, of which 44 were from North Carolina, 6 from South Carolina and 1 from Virginia. Molecular characterisation was performed on these samples by DNA sequencing on the ribosomal DNA 18S, ITS and 28S D2/D3. Species-specific primers were developed to identify turfgrass root-knot nematode through simplex or duplex PCR. Four species were identified, including *M*. *marylandi* Jepson & Golden in Jepson, 1987, *M*. *graminis* (Sledge & Golden, 1964) Whitehead, 1968, *M*. *incognita* (Kofoid & White, 1919) Chitwood, 1949 and *M*. *naasi* Franklin, 1965 through a combined analysis of DNA sequencing and PCR by species-specific primers. *M*. *marylandi* has been reported from North Carolina and South Carolina for the first time. Molecular diagnosis using PCR by species-specific primers provides a rapid and cheap species identification approach for turfgrass root-knot nematodes.

## Introduction

Turfgrasses are used worldwide for lawns of home and office buildings, athletic fields, other recreational facilities, and roadsides. In the United States, there are more than 50,000,000 lawns and 16,000 golf courses and the turfgrass area was estimated to be 30 million acres in 2007 [1,2]. In North Carolina (NC), there are 664 golf courses [http://www.golflink.com/golf-courses/state.aspx?state=NC] and the turfgrass industry is a 2.3 billion dollar a year industry (http://www.golf2020.com/media/32940/nc_golf_full_rpt_sri_final_29apr2013.pdf). However, maintenance of turfgrass is very challenging due to damage by various pests, including nematodes. During a survey from 2010 to 2013, 29 species of plant-parasitic nematodes belonging to 22 genera in 15 families were found associated with bermudagrass (*Cynodon dactylon*), creeping bentgrass (*Agrostis stolonifera*), and zoysiagrass (*Zoysia japonica*) in NC and South Carolina (SC) [3]. Of the plant-parasitic nematodes found in the survey, *Belonolaimus longicaudatus*, *Cactodera* sp. *Dolichodorus heterocephalus*, *Hemicycliophora* spp., *Hoplolaimus galeatus*, *Meloidogyne* spp., *Mesocriconema* spp., *Paratrichodorus* spp., and *Xiphinema* spp. were considered as damaging species [4]. Root-knot nematodes (RKN) ranked the third in prevalence after ring and spiral nematodes. They were recovered from about half of the samples and the population level was 131 ± 195 (10–1,160) per 500 cm^3^ soil [4].

RKNs are the most economically damaging plant-parasitic nematodes on horticultural and field crops. They are distributed worldwide and are obligate parasites of the roots of thousands of plant species, including monocotyledonous and dicotyledonous, herbaceous and woody plants. Symptoms associated with RKN infection include root galls, shoot chlorosis, stunted growth, nutrient deficiencies, and secondary infections by other pathogens [5]. A high level of damage can lead to total crop loss. RKNs have been recognized as economically damaging parasites associated with turfgrasses [6,7]. So far, there are nine species of *Meloidogyne* associated with turfgrasses worldwide, including *M*. *chitwoodi* Golden, O’Bannon, Santo & Finley, 1980, *M*. *fallax* Karssen, 1996, *M*. *graminicola* Golden & Birchfield, 1965, *M*. *graminis* (Sledge & Golden, 1964) Whitehead, 1968, *M*. *incognita* (Kofoid & White, 1919) Chitwood, 1949, *M*. *marylandi* Jepson & Golden in Jepson, 1987, *M*. *microtyla* Mulvey, Townshend & Potter, 1975, *M*. *minor* Karssen, Bolk, van Aelst, van den Beld, Kox, Korthals, Molendijk, Zilstra, van Hoof & Cook, 2004, and *M*. *naasi* Franklin, 1965 [6–12]. All but one species (*M*. *microtyla*) [10] were reported from turfgrasses in the United States [6,7]. In a survey of 238 golf courses in 10 states of the western United States, *M*. *naasi*, *M*. *marylandi*, *M*. *graminis*, *M*. *minor* and *M*. *chitwoodi* were identified, and they were considered as an important threat to turfgrasses [7].

Identification of RKN species is becoming increasingly important for the design of effective nematode management practices such as crop rotation and plant resistance. RKN species are normally identified using morphological features and morphometrics on the second-stage juveniles (J2), male morphology such as the form of the labial region, stylet and basal knobs, on the perineal patterns of mature females, differential host test or isozyme phenotyping of females [5,13]. With the number of described species over a hundred [5], the value of many of these characters becomes very questionable, often showing large intraspecific variation. Ideally, a diagnostic technique should not be limited to the availability of a particular developmental stage (eggs, juveniles or adults) and should require a small number of individuals to provide reliable identification of a species within a short period of time. The PCR process meets these requirements since it allows amplification of minute quantities of DNA which can be extracted from single nematodes, eggs or juveniles. As the J2 stage of RKN is readily available from soil in any assay lab, identifying this stage is most applicable for making species identification. Several molecular methods, such as the use of restriction-length polymorphisms (RFLP), random-amplified-polymorphic DNA (RAPD), satellite-DNA probes, sequence-characterised-amplified regions (SCAR), high-resolution-melting-curve analysis, real-time-PCR assays and DNA sequencing have become available for RKN identification [14–20]. However, few of these methods enable the identification of several RKN from turfgrasses and little is known about the RKN from turfgrass in the Southeastern United States.

The Nematode Assay Laboratory of the Agronomic Division of North Carolina Department of Agriculture & Consumer Services (NCDA&CS) is a high-throughput and publicly operated lab. In fiscal year 2012, 1,939 turfgrass samples were analyzed, accounting for 5.68% of the total samples and RKN was recovered from 13.72% of these turfgrass samples. The average population level of the second-stage juveniles was 97 per 500 cm^3^ soil and the highest population was 2,622. Turfgrass damage by root-knot nematodes is usually underestimated, due to the frequent grass-mowing and invisible galls on the small root system comparing to field crops. The objective of this study was to characterize the DNA sequences of RKNs on the ribosomal DNA 18S, ITS and 28S D2/D3, then to develop and validate turfgrass RKN species-specific primers for a reliable and rapid PCR assay to support our diagnostic services and to allow species identification of RKNs through a combined analysis of DNA sequencing and PCR by species-specific primers. The specificity and application of the assay were demonstrated.

## Materials and Methods

### Nematode samples

A total of 51 RKN populations from turfgrasses were tested in this study, which comprised of 44 from NC, 6 from SC and 1 from Virginia (Va) (Table 1). These samples were submitted to the Nematode Assay Laboratory of the Agronomic Division, NCDA&CS voluntarily from golf courses, sod farms and homeowners’ lawns. Some of the samples were collected during a plant-parasitic nematode survey of 111 golf courses in 39 counties in NC and SC in the summer 2011 [3]. No specific permissions were required in sampling for plant-parasitic nematodes and no endangered or protected species were involved. In addition, nine non-turfgrass nematode populations belonging to *M*. *arenaria* (Neal, 1889) Chitwood, 1949, *M*. *chitwoodi*, *M*. *enterolobii* Yang & Eisenback, 1983, *M*. *hapla* Chitwood, 1949, *M*. *incognita* and *M*. *javanica* (Treub, 1885) Chitwood, 1949 were included as reference species (sources in Table 1). The identification of these reference species had already confirmed by DNA sequencing and PCR by species-specific primers in other projects (data not shown herein). Nematodes were extracted from soil samples by a combination of elutriation [21] and centrifugation [22] methods. The nematode sample was poured into a counting dish (7.5 cm L × 3 cm W × 1.5 cm H) and the nematodes were identified and counted under a Nikon Diaphot 200 inverted microscope (Tokyo, Japan). Further species confirmation was performed with a Leica DM2500 compound microscope (Leica Microsystems Inc., Buffalo Grove, IL, USA) with interference contrast at up to 1,000× magnification.

**Table 1:** Species and isolates of root-knot nematodes sequenced in the present study.

DNA ID	Sample Lab ID	Nematode Species	Host	Locality (County, State)	18S + ITS GenBank Accession No.	28S D2/D3 GenBank Accession No.
1	08–23912	*M*. *marylandi*	Bermudagrass	Nash, NC	KP901041	KP901066
2	11–29600	*M*. *marylandi*	Bentgrass	Wayne, NC	KP901042	KP901066
3	11–29648	*M*. *marylandi*	Bentgrass	Wayne, NC	KP901042	KP901066
4	11–30359	*M*. *marylandi*	Bermudagrass	Lexington, SC	KP901043	KP901066
5	11–30365	*M*. *graminis*	Bermudagrass	Kershaw, SC	T44 KJ934143	KP901067
6	11–30368	*M*. *graminis*	Bermudagrass	Richland, SC	KP901044	
7	11–30383	*M*. *naasi*	Bentgrass	Avery, NC	T51 KJ934133	KP901068
8	11–30385	*M*. *naasi*	Bentgrass	Avery, NC	T53 KJ934132	KP901069
9	11–30669	*M*. *marylandi*	Bermudagrass	Beaufort, SC	KP901042	KP901066
10	11–30750	*M*. *marylandi*	Bermudagrass	Horry, SC	KP901042	KP901066
11	12–44	*M*. *naasi*	Bentgrass	Greenville, SC	T74 KJ934151	
12	12–419	*M*. *incognita*	Bermudagrass	Brunswick, NC		
13	12–502	*M*. *incognita*	Zoysiagrass	Moore, NC	KP901045	KP901070
14	12–10150	*M*. *incognita*	St. Augustine grass	New Hanover, NC		KP901071
15	12–30983	*M*. *marylandi*	Bermudagrass	Brunswick, NC	KP901042	KP901066
16	12–32498	*M*. *marylandi*	Zoysia grass	New Hanover, NC	KP901042	KP901066
17	12–32501	*M*. *marylandi*	Bermudagrass	New Hanover, NC	KP901042	KP901066
18	13–297	*M*. *marylandi*	Turfgrass	New Hanover, NC	KP901042	KP901066
19	13–509	*M*. *incognita*	Bermudagrass	Cumberland, NC	KP901046	KP901072
20	13–34030	*M*. *marylandi*	Zoysia grass	Brunswick, NC		KP901066
21	14–1424	*M*. *graminis*	Turfgrass	New Hanover, NC	KP901047	
22	14–13539	*M*. *naasi*	Turfgrass	VA	KP901048	KP901073
23	14–13931	*M*. *marylandi*	Zoysiagrass	New Hanover, NC	KP901043	KP901066
24	14–14302	*M*. *marylandi*	Turfgrass	New Hanover, NC	KP901042	KP901066
25	14–27025	*M*. *marylandi*	Bentgrass	Brunswick, NC	KP901042	KP901066
26	14–34425	*M*. *marylandi*	Bermudagrass	Moore, NC	KP901042	KP901066
27	14–35854	*M*. *marylandi*	Bermudagrass	Greene, NC	KP901049	KP901066
28	14–36555	*M*. *marylandi*	Turfgrass	Sampson, NC	KP901042	KP901066
29	14–36556	*M*. *marylandi*	Turfgrass	Sampson, NC	KP901042	KP901066
30	14–36570	*M*. *graminis*	Turfgrass	Sampson, NC	KP901050	KP901074
31	14–36577	*M*. *marylandi*	Turfgrass	Sampson, NC	KP901042	KP901066
32	14–37838	*M*. *marylandi*	Bentgrass	New Hanover, NC	KP901042	KP901066
33	14–39813	*M*. *graminis*	Bermudagrass	Brunswick, NC	KP901051	KP901075
34	14–39863	*M*. *marylandi*	Bermudagrass	Brunswick, NC	KP901042	KP901066
35	14–40153	*M*. *marylandi*	Bermudagrass	New Hanover, NC	KP901042	KP901066
36	14–41460	*M*. *graminis*	Bermudagrass	Brunswick, NC	KP901052	
37	14–41535	*M*. *marylandi*	Bermudagrass	Moore, NC	KP901042	KP901066
38	14–41641	*M*. *marylandi*	Turfgrass	Sampson, NC	KP901042	KP901066
39	14–41755	*M*. *incognita*	Bentgrass	Mecklenburg, NC	KP901045	KP901071
40	15–565	*M*. *graminis*	St. Augustine grass	New Hanover, NC	KP901053	
41	15–570	*M*. *graminis*	Bentgrass	Mecklenburg, NC		KP901066
42	15–1105	*M*. *marylandi*	Bermudagrass	Guilford, NC	KP901042	KP901066
43	15–2102	*M*. *marylandi*	Turfgrass	Moore, NC	KP901042	KP901066
44	15–2131	*M*. *graminis*	Bentgrass	Mecklenburg, NC	KP901054	KP901067
45	15–2170	*M*. *marylandi*	Turfgrass	Sampson, NC	KP901042	
46	15–4651	*M*.*graminis*	Zoysiagrass	New Hanover, NC	KP901055	KP901067
47	15–4652	*M*.*graminis*	Centipedgrass	New Hanover, NC	KP901055	KP901067
48	15–5634	*M*.*graminis*	Fescue	Wake, NC		KP901076
49	15–9785	*M*. *marylandi*	Turfgrass	Moore, NC	KP901042	KP901066
50	15–10906	*M*. *marylandi*	Turfgrass	Mecklenburg, NC	KP901042	
51	15–11834	*M*. *graminis*	Fescue	Wake, NC	KP901056	KP901077
52	12–31829	*M*. *incognita*	Peach	Moore, NC	KP901057	KP901078
53	13–639	*M*. *enterolobii*	Soybean	Johnston, NC	KP901058	KP901079
54	15–7996	*M*. *chitwoodi*	Potato	TX	KP901059	KP901080
55	15–26571	*M*. *incognita*	Tobacco	Graham, NC	KP901060	KP901081
56	GuMa	*M*. *arenaria*	Unknown	China	KP901061	KP901082
57	GuMj	*M*. *javanica*	Unknown	China	KP901062	KP901083
58	VW4	*M*. *javanica*	Unknown	USA	KP901063	KP901084
59	VW6	*M*. *incognita*	Unknown	USA	KP901064	KP901085
60	VW9	*M*. *hapla*	Unknown	USA	KP901065	KP901086

#### DNA extraction

For molecular analysis, a single or up to 10 nematodes of the J2 from the same sample were hand-picked into 10-μl AE buffer (10 mM Tris-Cl, 0.5 mM EDTA; pH 9.0) on a glass microscope slide (7.5 cm x 2.5 cm). The nematodes were then macerated with a pipette tip into pieces and collected in 50-μl AE buffer and stored at -20°C.

#### DNA amplification, cleaning and sequencing

**Fig 1:**
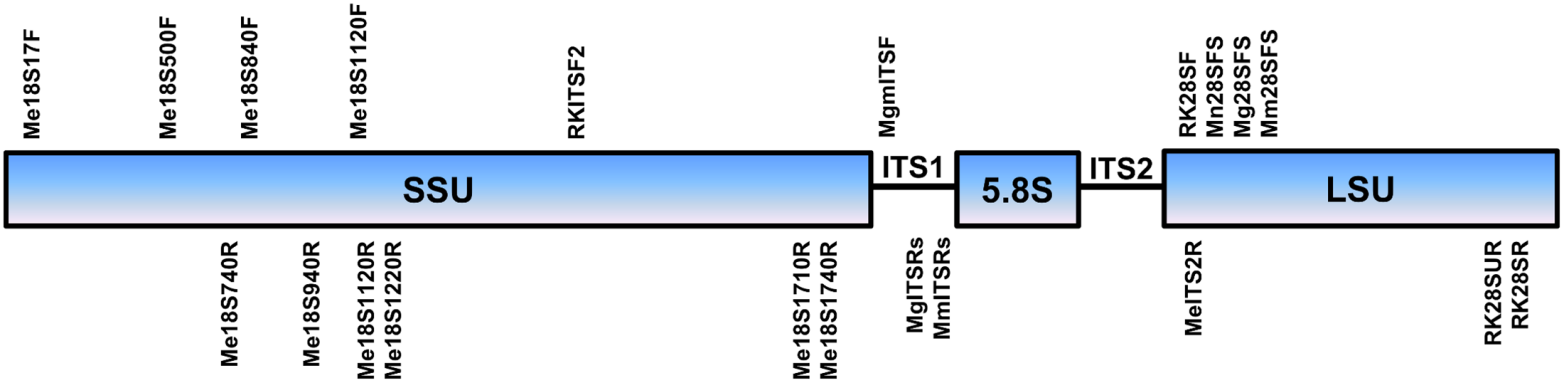
Prime map for PCR amplification and DNA sequencing on ribosomal DNA of *Meloidogyne* species. Primers on the top are the forward primers and primers on the bottom are the reverse primers.

The primers used for PCR and DNA sequencing are given in Table 2. The primers SSUF07/SSUR26 [23], 18S965/18S1573R [24], and 18SnF/18SnR [25] were used to amplify the ribosomal DNA near-full-length 18S gene. The primers rDNA2/ rDNA1.58S [26,27] were used to amplify the ITS1 rDNA region. The primers D2a/D3b [28] were used to amplify the partial rDNA 28S gene D2/D3 domain. PCR for these genes was also conducted using various combinations of universal forward and reverse primers designed for *Meloidogyne* to ensure high success in PCR (Table 2). These primers were based on the conserved sites from a multiple alignment of many representative *Meloidogyne* species from the GenBank and their approximate positions are shown in Fig 1. The primer selection criteria were as follows: Tm (melting temperature) 55 to 60°C, primer length 18 to 22 bp, and absence of secondary structure when possible. These primers were synthesized by Integrated DNA Technologies, Inc. (Coralville, Iowa, USA). The 25-μl PCR was performed using 12.5-μl 2X Apex Taq red master mix DNA polymerase (Genesee Scientific Corporation, San Diego, CA, USA), 9.5-μl water, 1-μl each of 10-μM forward and reverse primers, and 1μl of DNA template according to the manufacturer’s protocol in a Veriti^®^ thermocycler (Life Technologies, Carlsbad, CA, USA). The thermal cycler program for PCR was as follows: denaturation at 95°C for 5 min, followed by 40 cycles of denaturation at 94°C for 30 s, annealing at 55°C for 45 s, and extension at 72°C for 1 min. A final extension was performed at 72°C for 10 min. PCR products were cleaned using ExoSap-IT (Affymetrix, Inc., Santa Clara, CA, USA) according to the manufacturer’s protocol. DNA sequencing was performed using PCR primers for direct sequencing by dideoxynucleotide chain termination using an ABI PRISM BigDye terminator cycle sequencing ready reaction kit (Life Technologies, Carlsbad, CA, USA) in an Applied Biosystems 3730 XL DNA Analyzer (Life Technologies) by the Genomic Sciences Laboratory (North Carolina State University, Raleigh, NC, USA). The molecular sequences were compared with other nematode species available at the GenBank sequence database using the BLASTn homology search program.

**Table 2:** Primers used for polymerase chain reaction and DNA sequencing.

Primer	Gene	Sequence (5’ to 3’)	Reference
SSUF07	18S	AAAGATTAAGCCATGCATG	23
SSUR26	18S	CATTCTTGGCAAATGCTTTCG	23
18S965	18S	GGCGATCAGATACCGCCCTAGTT	24
18S1573R	18S	TACAAAGGGCAGGGACGTAAT	24
18SnF	18S	TGGATAACTGTGGTAATTCTAGAGC	25
18SnR	18S	TTACGACTTTTGCCCGGTTC	25
rDNA2	ITS	TTGATTACGTTCCCTGCCCTTT	26
rDNA1.58S	ITS	ACGAGCCGAGTGATCCACCG	27
D2a	28S D2/D3	ACAAGTACCGTGAGGGAAAGT	28
D3b	28S D2/D3	TGCGAAGGAACCAGCTACTA	28
Inc–K14-F	SCAR	CCCGCTACACCCTCAACTTC	33
Inc–K14-R	SCAR	GGGATGTGTAAATGCTCCTG	33
MR	28S D2/D3	AACCGCTTCGGACTTCCACCAG	34
Me18S17F	18S	GAGAAACCGCGAACGGCTCA	This study
Me18S500F	18S	GCAAGTCTGGTGCCAGCAGC	This study
Me18S740R	18S	TCCATGCACGATCATTCAAGCG	This study
Me18S840F	18S	ATTTGTATGGTCCCGTGAGAGG	This study
Me18S940R	18S	TGATCGCCTTCGAACCTCTG	This study
Me18S1120F	18S	ACCACCAGGAGTGGAGCC	This study
Me18S1120R	18S	GGCTCCACTCCTGGTGGT	This study
Me18S1220R	18S	ATGCACCACCATCCACTGAATC	This study
Me18S1710R	18S	GCCCGGTTCAAGCCACTG	This study
Me18S1740R	18S	GCAGGTTCACCTACAGCTACCT	This study
RK28SF	28S D2/D3	CGGATAGAGTCGGCGTATC	This study
RK28SR	28S D2/D3	GATGGTTCGATTAGTCTTTCGCC	This study
RKITSF2	ITS	GTAGGTGAACCTGCTGCTG	This study
MeITS2R	ITS	ATGCTTAAGTTCAGCGGGTG	This study
RK28SUR	28S D2/D3	CCCTATACCCAAGTCAGACGAT	This study
Mn28SFs	28S D2/D3	GTCTGATGTGCGACCTTTCACTAT	This study
Mm28SFs	28S D2/D3	GATGTGCGATATTTTTTTTTCGAA	This study
Mg28SFs	28S D2/D3	GATGTGCGATATTTTCCGTCAAGG	This study
MgmITSF	ITS	GATCGTAAGACTTAATGAGCC	This study
MgITSRs	ITS	TGCATAAGGCAACATAATGT	This study
MmITSRs	ITS	CTGATCTGATTTACATTACACGG	This study

#### Phylogenetic analyses

DNA sequences were edited with ChromasPro1.5 2003–2009 (Technelysium Pty Ltd, Helensvale, Australia) and aligned using ClustalW (http://workbench.sdsc.edu; Bioinformatics and Computational Biology group, Dept. Bioengineering, UC San Diego, CA, USA). The sequences used in phylogenetic analysis were chosen from the highest match based on BlastN result in GenBank against the four RKN species recovered from this study. The model of base substitution in the DNA sequence data was evaluated using MODELTEST version 3.06 [29]. The Akaike-supported model [30], the proportion of invariable sites, and the gamma distribution shape parameters and substitution rates were used in phylogenetic analyses using DNA sequence data. Bayesian analysis was performed to confirm the tree topology for each gene separately using MrBayes 3.1.0 [31], running the chain for 1,000,000 generations and setting the ‘burnin’ at 1,000. Markov Chain Monte Carlo (MCMC) methods were used within a Bayesian framework to estimate the posterior probabilities (pp) of the phylogenetic trees [32] using the 50% majority-rule. The λ2 test for homogeneity of base frequencies and phylogenetic trees was performed using PAUP* version 4.0 (Sinauer Associates, Inc. Publishers, Sunderland, MA, USA).

### Simplex PCR by species-specific primers

The species identification of *M*. *incognita* was confirmed using PCR by species-specific SCAR primers Inc-K14-F/Inc-K14-R which produce a 399-bp DNA fragment [33]. Mn28SFs/RK28SUR in 28S D2/D3 were designed specific for *M*. *naasi* producing a 272-bp DNA fragment based on JN019291. Primers Mg28SFs/RK28SUR and Mm28SFs/RK28SUR in 28S D2/D3 were designed specific for *M*. *graminis* and *M*. *marylandi*, both producing a 198-bp DNA fragment based on JN019339 and JN019359 respectively. Primers MgmITSF/MgITSRs and MgmITSF/MmITSRs in ITS were designed specific for *M*. *graminis* and *M*. *marylandi*, producing a 267-bp based on JN241882 and 323-bp DNA fragment based on JN157855 respectively. Universal primers RK28SF/MR (MR is from Hu et al. [34]) in 28S D2/D3 were designed for all *Meloidogyne* species producing a 612-bp DNA fragment based on JN019339 as an internal positive control for all assays. A DNA sample was prepared from a mixture of four species (DNA ID: 2, 19, 22 and 48 in Table 1) to test the scenario if a mixed species was present. The PCR condition is the same as described above.

### Duplex PCR by ITS species-specific primers and 28S universal primers

The 25-μl duplex PCR was performed using 12.5-μl 2X Apex Taq red master mix DNA polymerase, 7.5-μl water, 1-μl each of 10-μM forward and reverse primers specific for *M*. *graminis* and *M*. *marylandi*, plus 1-μl each of 10-μM primers RK28SF/MR as internal positive control, and 1-μl of DNA template. The PCR condition is the same as described above.

## Results and Discussion

### Root-knot nematode identification

The J2s of RKNs were recovered from the turfgrass soil samples. Species identification in this study was based on the combined analysis of DNA sequencing on the rDNA 18S, ITS and 28S D2/D3 (Table 1) and PCR by species-specific primers (Table 3). Four species were recovered including *M*. *marylandi*, *M*. *graminis*, *M*. *incognita* and *M*. *naasi*; the results are given in Table 1.

**Table 3:** Nematode simplex and/or duplex PCR results.

Species	DNA ID			Species-specific primers				Internal positive control
Primer		Mg28SFs/RK28SUR	MgmITSF/MgITSRs	Mm28SFs/RK28SUR	MgmITSF/MmITSRs	Mn28SFs/RK28SUR	Inc-K14-F/Inc-K14-R	RK28SF/MR
Specific species		*M*. *gramini s*	*M*. *graminis*	*M*. *marylandi*	*M*. *marylandi*	*M*. *naasi*	*M*. *incognita*	*Meloidogyne* spp.
PCR amplicon size		198bp	267bp	198bp	323bp	272bp	399bp	612bp
GenBank # for determining PCR amplicon size		JN019339	JN241882	JN019359	JN157855	JN019291		JN019339
Target gene		28S D2/D3	ITS	28S D2/D3	ITS	28S D2/D3	SCAR	28S D2/D3
*M*. *arenaria*	56	**-**	**-**	**-**	**-**	**-**	**-**	**+**
*M*. *chitwoodi*	54	**-**	**-**	**-**	**-**	**-**	**-**	**+**
*M*. *enterolobii*	53	**-**	**-**	**-**	**-**	**-**	**-**	**+**
*M*. *graminis*	30	**+**	**+**	**-**	**-**	**-**	**-**	**+**
*M*. *graminis*	36	**+**	**+**	**-**	**-**	**-**	**-**	**+**
*M*. *graminis*	40	**+**	**+**	**-**	**-**	**-**	**-**	**+**
*M*. *graminis*	41	**+**	**+**	**-**	**-**	**-**	**-**	**+**
*M*. *graminis*	44	**+**	**+**	**-**	**-**	**-**	**-**	**+**
*M*. *graminis*	46	**+**	**+**	**-**	**-**	**-**	**-**	**+**
*M*. *graminis*	47	**+**	**+**	**-**	**-**	**-**	**-**	**+**
*M*. *graminis*	48	**+**	**+**	**-**	**-**	**-**	**-**	**+**
*M*. *graminis*	51	**+**	**+**	**-**	**-**	**-**	**-**	**+**
*M*. *hapla*	60	**-**	**-**	**-**	**-**	**-**	**-**	**+**
*M*. *incognita*	13	**-**	**-**	**-**	**-**	**-**	**+**	**+**
*M*. *incognita*	19	**-**	**-**	**-**	**-**	**-**	**+**	**+**
*M*. *incognita*	52	**-**	**-**	**-**	**-**	**-**	**+**	**+**
*M*. *incognita*	55	**-**	**-**	**-**	**-**	**-**	**+**	**+**
*M*. *incognita*	59	**-**	**-**	**-**	**-**	**-**	**+**	**+**
*M*. *javanica*	57	**-**	**-**	**-**	**-**	**-**	**-**	**+**
*M*. *javanica*	58	**-**	**-**	**-**	**-**	**-**	**-**	**+**
*M*. *marylandi*	1	**-**	**-**	**+**	**+**	**-**	**-**	**+**
*M*. *marylandi*	2	**-**	**-**	**+**	**+**	**-**	**-**	**+**
*M*. *marylandi*	10	**-**	**-**	**+**	**+**	**-**	**-**	**+**
*M*. *marylandi*	20	**-**	**-**	**+**	**+**	**-**	**-**	**+**
*M*. *marylandi*	23	**-**	**-**	**+**	**+**	**-**	**-**	**+**
*M*. *marylandi*	24	**-**	**-**	**+**	**+**	**-**	**-**	**+**
*M*. *marylandi*	25	**-**	**-**	**+**	**+**	**-**	**-**	**+**
*M*. *marylandi*	26	**-**	**-**	**+**	**+**	**-**	**-**	**+**
*M*. *marylandi*	27	**-**	**-**	**+**	**+**	**-**	**-**	**+**
*M*. *marylandi*	29	**-**	**-**	**+**	**+**	**-**	**-**	**+**
*M*. *marylandi*	31	**-**	**-**	**+**	**+**	**-**	**-**	**+**
*M*. *marylandi*	38	**-**	**-**	**+**	**+**	**-**	**-**	**+**
*M*. *marylandi*	42	**-**	**-**	**+**	**+**	**-**	**-**	**+**
*M*. *marylandi*	43	**-**	**-**	**+**	**+**	**-**	**-**	**+**
*M*. *marylandi*	50	**-**	**-**	**+**	**+**	**-**	**-**	**+**
*M*. *naasi*	22	**-**	**-**	**-**	**-**	**+**	**-**	**+**

### DNA sequencing

The rDNA 18S, ITS and 28S D2/D3 were successfully sequenced; their accession numbers from the GenBank are presented in Table 1. A lot of the sequences are identical, but sequence variations were observed in each gene among some populations. The 2,015-bp DNA sequences of 18S and ITS of *M*. *marylandi* (KP901041 and KP901042) are identical to a sequence of *M*. *marylandi* from the GenBank (JN241856) in 18S region and 1-3-bp differences with other populations of *M*. *marylandi* (KP901043 and KP901049). DNA sequences of 18S of *M*. *graminis* are represented by KJ934143, KP901044, KP901047, and KP901050- KP901056. They have 0–23-bp differences in 1,737-bp 18S and are the closest to sequences of *M*. *marylandi*. Two 2,015-bp DNA sequences of 18S and ITS sequences of *M*. *naasi* (KJ934132 and KJ934133) are identical and have 2-bp differences from KP901048. These sequences have the highest match with two 18S sequences of *M*. *naasi* from the GenBank (AY593901 and AY593902). The 2,020-bp DNA sequences of 18S and ITS of *M*. *incognita* (KP901046) has 6-bp differences with a reference species *M*. *incognita* (KP901064). It has 99% identity on 18S with the tropical root-knot nematodes, including *M*. *incognita* (AY268120, AY284621, AY942624), *M*. *arenaria* (AY942623), *M*. *javanica* (AY268121, EU669938) and *M*. *floridensis* (AY942621), which failed to differentiate these tropical species.

The DNA sequence of 28S D2/D3 (KP901066) on *M*. *marylandi* is fairly conserved; no sequence variation was observed among all populations. It has 1-bp difference with a sequence of *M*. *marylandi* (JN157852) from the GenBank. The DNA sequence of 28S D2/D3 (KP901067, KP901074 to KP901077) on *M*. *graminis* have 0-3-bp differences. They highly match with sequences of *M*. *graminis* from the GenBank (JN019327, JN019329, JN019331 and JN157850). The 985-bp DNA sequence (KP901066) of *M*. *marylandi* is close to *M*. *graminis* (KP901067), with 97% identity; the divergence is significant to differentiate these two sister species by this gene and thus this gene was chosen as a diagnostic marker in PCR. Three 987-bp DNA sequences of 28S D2/D3 of *M*. *naasi* (KP091068, KP901069 and KP901073) are identical. These sequences are also identical to the sequences of *M*. *naasi* from the GenBank (JN019265, JN019266, JN019299, JN019304, JN019312 and KC241979). The DNA sequence of 28S D2/D3 (KP901070, KP901071, KP901072 and KP901081) on *M*. *incognita* have 12-bp variable sites over 1,008-bp fragment. These sequences are also close to some reference tropical RKN species *M*. *incognita* (KP901078 and KP901085), *M*. *arenaria* (KP901082) and *M*. *javanica* (KP901083 and KP901084) with 99% identity. This result agreed with previous studies that rDNA is very conserved with high similarity among the three most common tropical RKN species, namely *M*. *incognita*, *M*. *arenaria* and *M*. *javanica* on 18S [7,35–38], ITS [7,39], 28S [7,39] and IGS [40]. Therefore, the conserved ribosomal DNA can’t separate these tropical RKNs. The mitochondrial DNA has a faster rate of evolution than the corresponding nuclear genes, creating sufficient nucleotide variation for species-level analyses [15]. The region of the mitochondrial genome flanked by the COII gene and the large (16S) ribosomal gene were successfully applied in large-scale regional RKN survey through PCR and RFLP [14]. Unfortunately, numerous attempts using the same primers [14] or designing new primers for turfgrass RKNs in this project were not successful, with a low rate of success in PCR and insufficient DNA sequence data to generate any meaningful results. Thus, the use of mitochondrial genome on molecular identification for turfgrass RKNs needs further study.

### Molecular phylogenetic relationships

**Fig 2:**
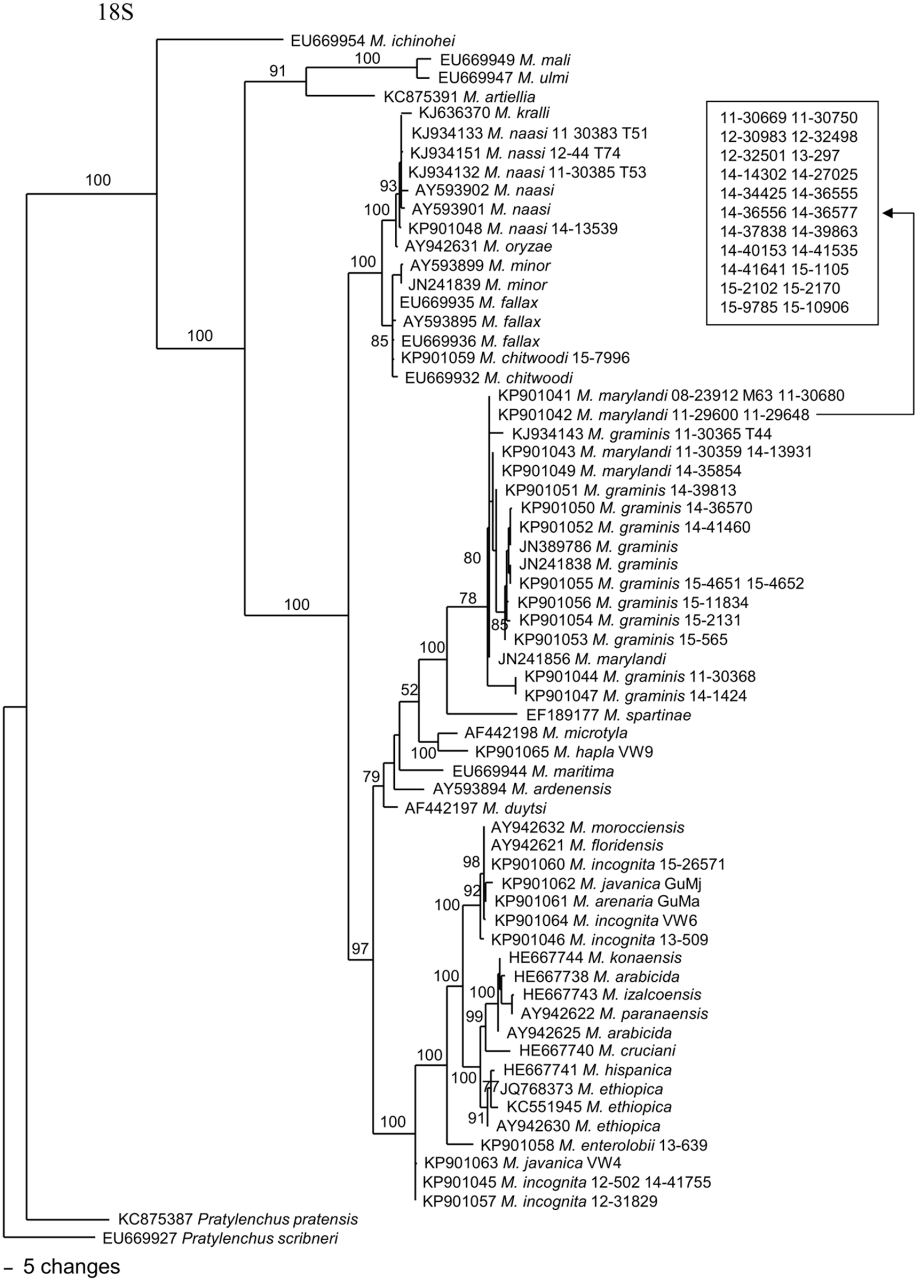
The 10001st Bayesian tree inferred from 18S under GTR+I+G model (-lnL = 7750.8096; AIC = 15521.6191; freqA = 0.2618; freqC = 0.2096; freqG = 0.2649; freqT = 0.2638; R(a) = 1.6485; R(b) = 3.5003; R(c) = 2.7135; R(d) = 0.5616; R(e) = 5.9174; R(f) = 1; Pinvar = 0.5043; Shape = 0.5848). Posterior probability values exceeding 50% are given on appropriate clades.

A phylogenetic tree based on the near-full-length 18S rDNA from a multiple alignment of 1,699 total characters is presented in Fig 2. This dataset has 1,443 constant characters (85.2%). Using two *Pratylenchus* species as outgroup taxa, this tree placed turfgrass nematodes in three distinct groups. *M*. *naasi* populations are in a clade with *M*. *kralli*, *M*. *oryzae*, *M*. *minor*, *M*. *fallax* and *M*. *chitwoodi*. *M*. *marylandi* and *M*. *graminis* populations are very closely related and are in a clade with *M*. *spartinae*. *M*. *incognita* populations are in a clade with tropical RKNs *M*. *arenaria*, *M*. *javanica*, *M*. *floridensis* and *M*. *morocciensis*. This tree generally agrees with the trees from McClure et al. [7] and a 18S-rDNA gene by Tigano et al. [36].

**Fig 3:**
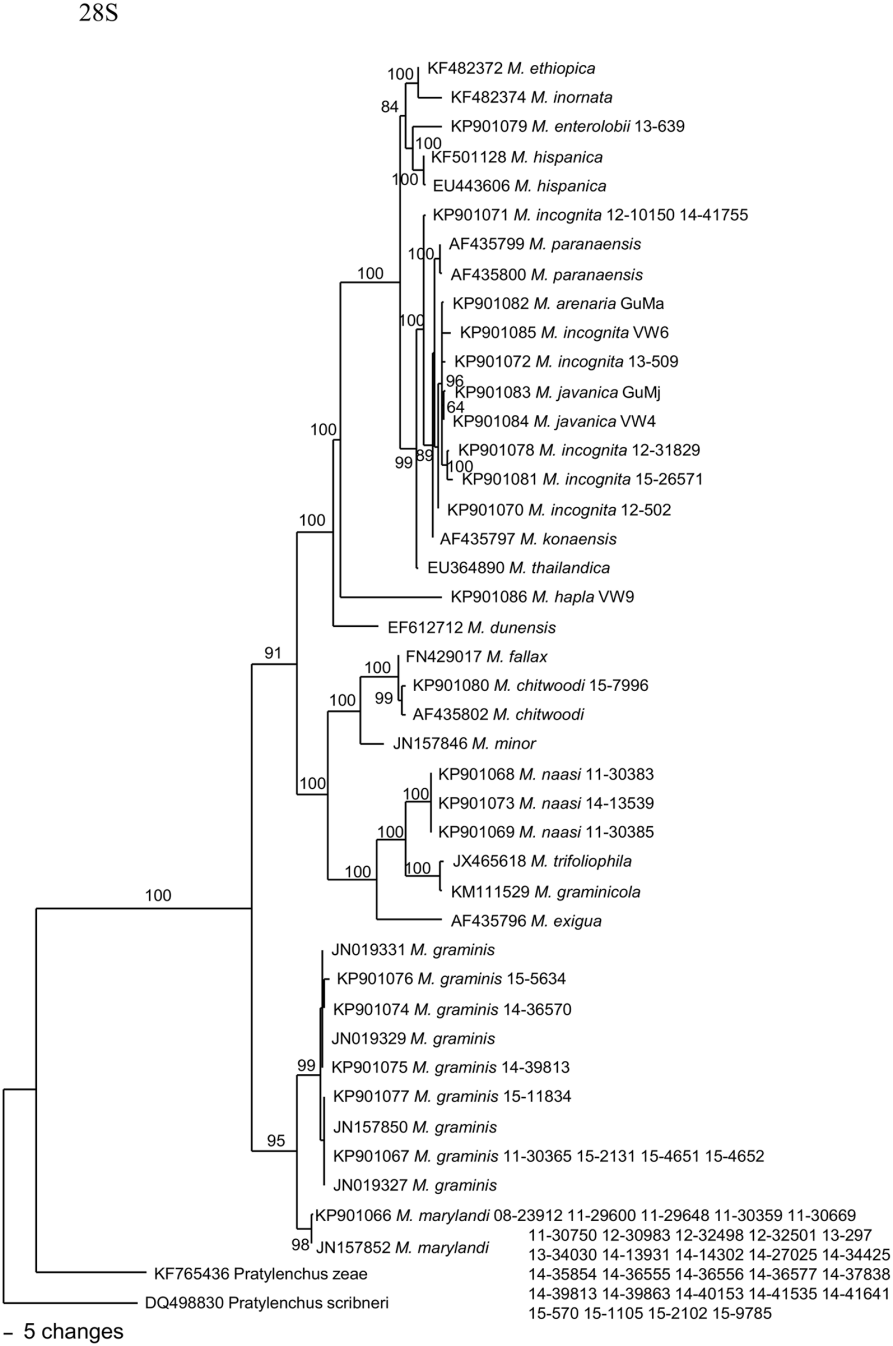
The 10001st Bayesian tree inferred from 28S D2/D3 under TVM+I+G model (-lnL = 4970.2949; AIC = 9958.5898; freqA = 0.2484; freqC = 0.1933; freqG = 0.2727; freqT = 0.2855; R(a) = 0.5776; R(b) = 2.9297; R(c) = 1.8072; R(d) = 0.228; R(e) = 2.9297; R(f) = 1; Pinvar = 0.2661; Shape = 0.6378) . Posterior probability values exceeding 50% are given on appropriate clades.

A phylogenetic tree based on the rDNA 28S D2/D3 sequences from a multiple alignment of 800 total characters is presented in Fig 3. This dataset has 596 constant characters (93.4%). Using two *Pratylenchus* species as outgroup taxa, this tree placed turfgrass nematodes in three distinct groups. *M*. *naasi* populations are in a clade with *M*. *trifoliophila*, *M*. *graminicola*, *M*. *exigua*, *M*. *minor* and *M*. *chitwoodi*; *M*. *marylandi* and *M*. *graminis* populations are in a monophyletic clade and are two distinct sister species. *M*. *incognita* populations are in a clade with *M*. *arenaria*, *M*. *javanica*, *M*. *paranaensis* and *M*. *konaensis*. This tree generally agrees with the trees from McClure et al. [7].

### Simplex PCR by species-specific primers

Results of simplex PCR by species-specific primers are given in Table 3. Using the internal positive control primer set RK28SF/MR, all assays are 100% positive which proves its usefulness for PCR on RKN. Primer sets Mg28SFs/RK28SUR and MgmITSF/MgITSRs are positive only for *M*. *graminis*. Primer sets Mm28SFs/RK28SUR and MgmITSF/MmITSRs are positive only for *M*. *marylandi*. Results of 28S primers and ITS primers agree with each other. Primer set Mn28SFs/RK28SUR are positive only for *M*. *naasi*. However, *M*. *naasi* is rather rare in this study and only one population (14–13539) was available for further PCR testing by specie-specific primers. Two other populations (11–30383 and 11–30385) are also sequenced on 28S D2/D3 and they are all identical with population 14–13539. Although these sequences are identical to the sequences of *M*. *naasi* from the GenBank (JN019265, JN019266, JN019299, JN019304, JN019312 and KC241979), more samples could be included to validate the specificity in the future. Primer set Inc-K14-F/ Inc-K14-R are positive only for *M*. *incognita*. Other reference species, including *M*. *arenaria*, *M*. *chitwoodi*, *M*. *enterolobii*, *M*. *hapla*, and *M*. *javanica*, are all negative to these species-specific primers, but all positive to positive control primer set RK28SF/MR.

**Fig 4:**
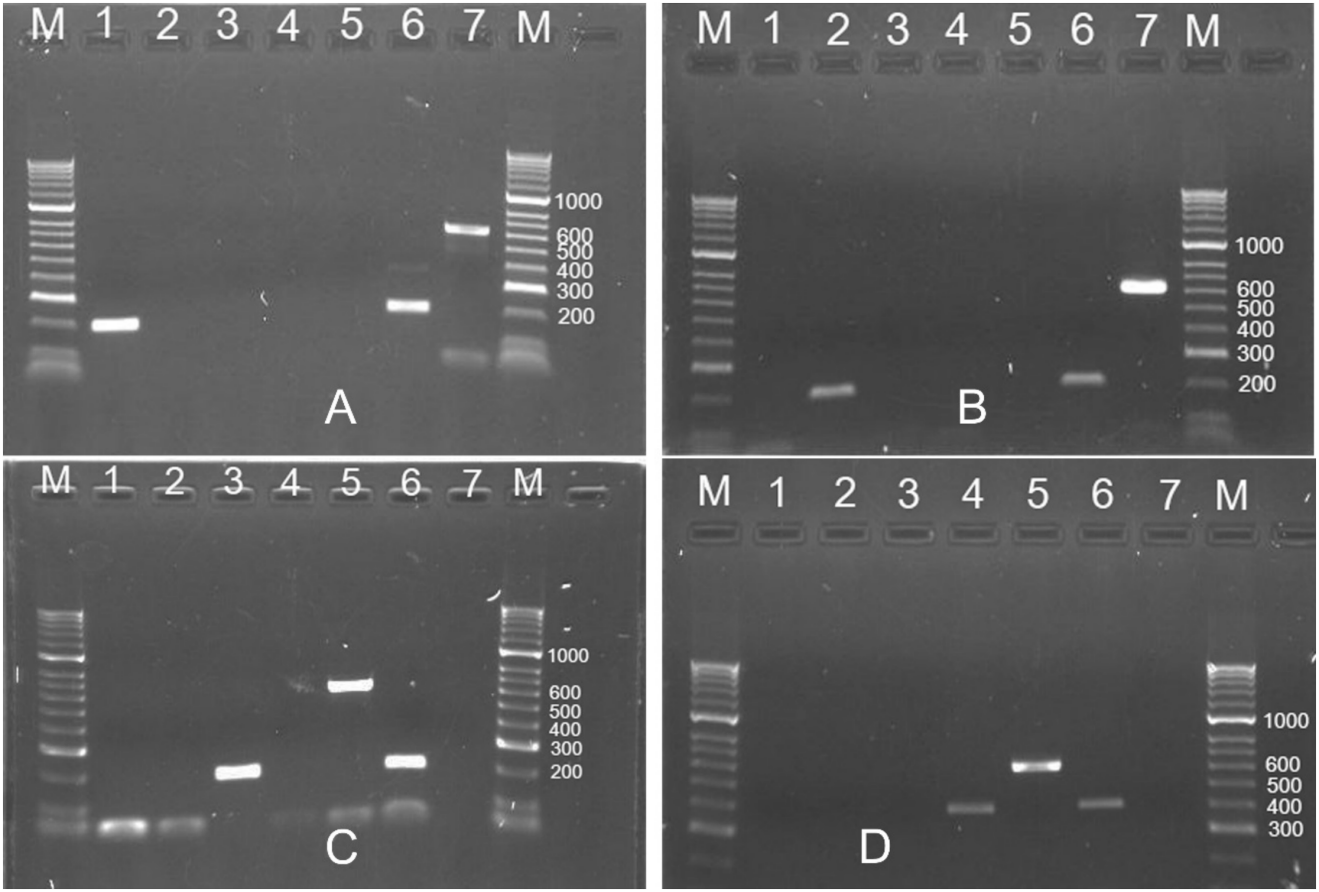
Simplex PCR results for four common *Meloidogyne* species from turfgrass using species-specific primers. A. Simplex PCR results using *M*. *graminis*-specific primers (Mg28SFs/RK28SUR). M: HyperLadder II DNA Marker; 1: *M*. *graminis* (DNA ID: 48); 2: *M*. *marylandi* (2); 3: *M*. *naasi* (22); 4: *M*. *incognita* (19); 5: Water; 6: Mixed DNA of four species (2, 19, 22 and 48); 7: *M*. *graminis* (48) (Universal primers, RK28SF/MR). B. Simplex PCR results using *M*. *marylandi*-specific primers (Mm28SFs/RK28SUR). 1: *M*. *graminis* (48); 2: *M*. *marylandi* (2); 3: *M*. *naasi* (22); 4: *M*. *incognita* (19); 5: Water; 6: Mixed DNA of four species (2, 19, 22 and 48); 7: *M*. *marylandi* (2) (Universal primers, RK28SF/MR). C. Simplex PCR results using *M*. *naasi*-specific primers (Mn28SFs/RK28SUR). 1: *M*. *graminis* (48); 2: *M*. *marylandi* (2); 3: *M*. *naasi* (22); 4: *M*. *incognita* (19); 5: *M*. *naasi* (22) (Universal primers, RK28SF/MR); 6: MixDNA of four species (2, 19, 22 and 48); 7: Water. D. Simplex PCR results using *M*. *incognita*-specific primers (Inc-K14-F/Inc-K14-R). 1: *M*. *graminis* (48); 2: *M*. *marylandi* (2); 3: *M*. *naasi* (22); 4: *M*. *incognita* (19); 5: *M*. *incognita* (19) (Universal primers, RK28SF/MR); 6: MixDNA of four species (2, 19, 22 and 48); 7: Water.

The simplex PCR results for testing four common *Meloidogyne* species from turfgrass using species-specific primers are presented in Fig 4. Fig 4A amplified a 198-bp DNA fragment in 28S D2/D3 using Mg28SFs/RK28SUR for *M*. *graminis*, but the other three species failed to get any PCR products. Fig 4B amplified a 198-bp DNA fragment in 28S D2/D3 using Mm28SFs/RK28SUR for *M*. *marylandi*, but the other three species failed to get any PCR products. Fig 4C amplified a 272-bp DNA fragment in 28S D2/D3 using Mn28SFs/RK28SUR for *M*. *naasi*, but the other three species failed to get any PCR products. Fig 4D amplified a 399-bp DNA fragment in SCAR using Inc-K14-F/Inc-K14-R for *M*. *incognita*, but the other three species failed to get any PCR products. All these four samples produced a 612-bp DNA fragment using RK28SF/MR. All results are positive if the DNA is from a mixture of four species. Water used as a negative control in all these assays was negative.

### Duplex PCR by ITS species-specific primers and 28S universal primers

**Fig 5:**
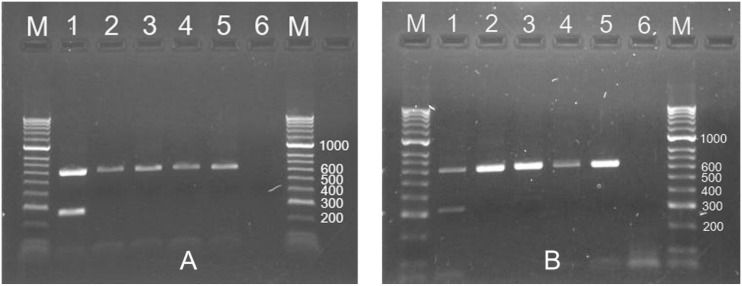
Duplex PCR results for two common *Meloidogyne* species from turfgrass using ITS species-specific primers plus 28S universal primers. A. Duplex PCR results using *M*. *graminis*-specific primers (MgmITSF/MgITSRs) plus RK28SF/MR. M: HyperLadder II DNA Marker; 1: *M*. *graminis* (DNA ID: 48); 2: *M*. *marylandi* (2); 3: *M*. *naasi* (22); 4: *M*. *incognita* (19); 5: *M*. *graminis* (48) (only RK28SF/MR); 6: Water. B. Duplex PCR results using *M*. *marylandi*-specific primers (MgmITSF/MmITSRs) plus RK28SF/MR. 1: *M*. *marylandi* (2); 2: *M*. *graminis* (48); 3: *M*. *naasi* (22); 4: *M*. *incognita* (19); 5: *M*. *marylandi* (2) (only RK28SF/MR); 6: Water.

Results of duplex PCR by ITS species-specific primers and 28S universal primers are given in Table 3 and agree with simplex PCR results. The duplex PCR results for testing two most common *Meloidogyne* species (*M*. *marylandi* and *M*. *graminis*) from turfgrass using ITS species-specific primers and 28S universal primers are presented in Fig 5. Fig 5A amplified a 267-bp DNA fragment using MgmITSF/MgITSRs and a 612-bp DNA fragment using RK28SF/MR for *M*. *graminis*, but the other three species only amplified a 612-bp DNA fragment by RK28SF/MR. Fig 5B amplified a 323-bp DNA fragment in ITS using MgmITSF/MmITSRs and a 612-bp DNA fragment using RK28SF/MR for *M*. *marylandi*, but the other three species only amplified a 612-bp DNA fragment by RK28SF/MR. Water used as a negative control in all these assays was negative. The duplex PCR provides any assay to detect the target species and any RKNs in a single reaction to prevent false negatives caused by failure of the PCR for any reason.

In conclusion, this study characterized DNA sequences on rDNA 18S, ITS and 28S D2/D3 on a wide range of RKN populations from turfgrasses mainly from NC. Universal primers were also developed for PCR on the genus *Meloidogyne* for these three gene fragments. Analysis of the sequences through BlastN search and phylogenetic analysis revealed four distinct species, namely *M*. *marylandi*, *M*. *graminis*, *M*. *incognita* and *M*. *naasi*, the first two being the predominant species in NC. This result is different from the western United States where *M*. *naasi* was determined to be the most common species [7,12]. In this same study [7,12],.*M*. *minor* was only detected from Washington and *M*. *chitwoodi* and *M*. *fallax* only from California, but none of these three species were detected in the current study.

*Meloidogyne marylandi* was found on bermudagrass (*Cynodon dactylon* (L.) Pers) in College Park, Maryland, USA and first described by Jepson & Golden [41]. In addition to Maryland, *M*. *marylandi* has been reported from Texas [42], Florida [43], Oklahoma [44] and the western United States, including Arizona, California, Nevada, Utah and Hawaii [7]. Outside the United States, *M*. *marylandi* has been found in Japan [45], Israel [46], and Costa Rica [47]. It feeds and reproduces on some turfgrasses, including bermudagrass [41,43,48] and zoysiagrass (*Zoysia matrella* Steud.) [45]. This is the first report of this species in NC and SC.

Species-specific primers on rDNA 28S D2/D3 were developed to identify turfgrass RKN through simplex PCR by species-specific primers on *M*. *marylandi*, *M*. *graminis* and *M*. *naasi*. Species-specific primers on ITS were also developed to identify two most common species *M*. *marylandi* and *M*. *graminis* to allow species confirmation using an additional marker through simplex or duplex PCR. SCAR primers Inc-K14-F/Inc-K14-R [33] were employed to identify *M*. *incognita* which produces a 399-bp DNA fragment. In addition, the RKN-universal primers RK28SF/MR were designed and included to amplify a 612-bp DNA fragment as a RKN endogenous control to detect the presence of RKN rDNA 28S gene, so that a RKN-negative sample can still be evaluated to exclude false negatives due to instrument, pipetting, reagent, and/or reaction failure. Compared with other molecular diagnosis [7,14,16,17,19,20,49], this assay only requires routine PCR and electrophoresis and is simple, cheap and rapid (<4 h), without further restriction digestion, DNA sequencing or expensive real-time PCR equipment and reagents. This molecular diagnosis using PCR by species-specific primers provides a rapid species identification approach for turfgrass RKN independent of morphology.

## References

[1] Emmons R (1995) Turfgrass Science and Management. 2nd edn Delmar, New York 512 p.

[2] Lyman GT, Throssell CS, Johnson ME, Stacey GA, Brown CD (2007) Golf course profile describes turfgrass, landscape and environmental stewardship features. Online. Appl Turfgrass Sci

[3] Zeng Y, Ye W, Tredway L, Martin S, Martin M (2012) Taxonomy and morphology of plant-parasitic nematodes associated with turfgrasses in North and South Carolina, USA. Zootaxa 3452:1–46.

[4] Ye W, Zeng Y, Tredway L, Martin S, Martin M (2012) Plant-parasitic nematodes in Carolina turfgrass. *Carolinas Green* (March/April): 26–28.

[5] Hunt D, Handoo ZA (2009) Taxonomy, identification and principal species In Root-knot Nematodes, Eds Perry, Moens, and Starr. Publisher: CABI Publishing: 55–97.

[6] Vandenbossche B, Viaene N, Sutter N, de Maes M, Karssen G, Bert W (2011) Diversity and incidence of plant-parasitic nematodes in Belgian turf grass. Nematology 13: 245–256.

[7] McClure MA, Nischwitz C, Skantar AM, Schmitt ME, Subbotin SA (2012) Root-knot nematodes in golf course greens of the western United States. Plant Dis 96: 635–647. 10.1094/PDIS-09-11-0808 30727525

[8] Sledge EB, Golden AM (1964) Hypsoperine graminis (Nematode: Heteroderidae), a new genus and species of plant parasitic nematode. Proc Helm Soc Wash 31:83–88.

[9] Franklin MT (1965) A root-knot nematode, Meloidogyne naasi n. sp., on field crops in England and Wales. Nematologica 11: 79–86.

[10] Mulvey RH, Townshend JL, Potter JW (1975) Meloidogyne microtyla sp. nov. from southwestern Ontario, Canada. Canadian J Zool 53(11): 1528–1536.

[11] Crow WT (2005) Plant-parasitic nematodes on golf course turf. Outlooks on Pest Management 16: 10–15.

[12] Nischwitz C, Skantar A, Handoo ZA, Hult MN, Schmitt ME, McClure MA (2013) Occurrence of *Meloidogyne fallax* in North America, and molecular characterization of *M*. *fallax* and *M*. *minor* from U.S. golf course greens. Plant Dis 97: 1424–1430. 10.1094/PDIS-03-13-0263-RE 30708461

[13] Eisenback JD (1985) Diagnostic characters useful in the identification of the four most common species of root-knot nematodes (*Meloidogyne* spp.) Pp. 95–112 in Sasser J.N. and Carter C.C., eds. An advanced treatise on *Meloidogyne*, vol. 1. Biology and control. North Carolina State University Graphics, Raleigh.

[14] Powers TO, Mullin PG, Harris TS, Sutton LA, Higgins RS (2005) Incorporating molecular identification of *Meloidogyne* spp. into a large-scale regional nematode survey. J Nematol 37: 226–235. 19262865 PMC2620951

[15] Blok VC, Powers TO (2009) Biochemical and molecular identification In Root-knot Nematodes, Eds Perry, Moens, and Starr. Publisher: CABI Publishing: 98–118.

[16] Holterman MHM, Oggenfuss M, Frey JE, Kiewnick S (2012) Evaluation of high-resolution melting curve analysis as a new tool for root-knot nematode diagnostics. J Phytopathol 160: 59–66.

[17] Adam MAM, Phillips MS, Blok VC (2005) Identification of *Meloidogyne* spp. from North East Libya and comparison of their inter-and intra-specific genetic variation using RAPDs. Nematology 7: 599–609.

[18] Zijlstra C, Donkers-Venne DTHM, Fargette M (2000) Identification of Meloidogyne incognita, M. javanica and M. arenaria using sequence characterised amplified region (SCAR) based PCR assays. Nematology 2: 847–853.

[19] Zeng Y, Ye W, Kerns J (2015) Molecular characterization and phylogenetic relationships of plant-parasitic nematodes associated with turfgrasses in North Carolina and South Carolina, USA. Plant Dis (In press). 10.1094/PDIS-10-14-1060-RE 30690976

[20] Berry SD, Fargette M, Spaull VW, Morand S, Cadet P (2008) Detection and quantification of root-knot nematode (Meloidogyne javanica), lesion nematode (Pratylenchus zeae) and dagger nematode (Xiphinema elongatum) parasites of sugarcane using real-time PCR. Mol Cellular Probes 22: 168–176. 10.1016/j.mcp.2008.01.003 18378423

[21] Byrd DW Jr., Barker KR, Ferris H, Nusbaum CJ, Griffin WE, Small RH, et al (1976) Two semi-automatic elutriators for extracting nematodes and certain fungi from soil. J Nematol 8: 206–212. 19308224 PMC2620186

[22] Jenkins WR (1964) A rapid centrifugal-floatation technique for separating nematodes from soil. Plant Dis Rep 48: 692.

[23] Floyd R, Abebe E, Papert A, Blaxter M (2002) Molecular barcodes for soil nematode identification. Mol Ecol 11: 839–850. 11972769 10.1046/j.1365-294x.2002.01485.x

[24] Mullin PG, Harris TS, Powers TO (2005) Phylogenetic relationships of Nygolaimina and Dorylaimina (Nematoda: Dorylaimida) inferred from small subunit ribosomal DNA sequences. Nematology 7: 59–79.

[25] Kanzaki N, Futai K (2002) A PCR primer set for determination of phylogenetic relationships of *Bursapelenchus* species within the *xylophilus* group. Nematology 4: 35–41.

[26] Vrain TC , Wakarchuk DA , Levesque AC , Hamilton RI (1992) Intraspecific rDNA restriction fragment length polymorphism in the *Xiphinema americanum* group. J Nematol 29:250–254.

[27] Cherry T, Szalanski AL, Todd TC, Powers TO (1997) The internal transcribed spacer region of Belonolaimus (Nemata: Belonolaimidae). J Nematol 29: 23–29. 19274130 PMC2619755

[28] Nunn GB (1992) Nematode molecular evolution. Ph.D. dissertation. University of Nottingham, UK.

[29] Posada D, Criandall KA (1998) Modeltest: testing the model of DNA substitution. Bioinformatics 14: 817–818. 9918953 10.1093/bioinformatics/14.9.817

[30] Arnold TW (2010) Uninformative parameters and model selection using Akaike's information criterion. J Wildlife Manage 74: 1175–1178.

[31] Huelsenbeck JP, Ronquist F (2001) Mr Bayes: Bayesian inference of phylogenetic trees. Bioinformatics 17: 1754–1755. 10.1093/bioinformatics/17.8.754 11524383

[32] Larget B, Simon DL (1999) Markov Chain Monte Carlo algorithms for the Bayesian analysis of phylogenetic trees. Mol Biol Evol 16: 750–759.

[33] Randig O, Bongiovanni M, Carneiro RMDG, Castagnone-Sereno P (2002) Genetic diversity of root-knot nematodes from Brazil and development of SCAR markers specific for the coffee-damaging species. Genome 45: 862–870. 12416618 10.1139/g02-054

[34] Hu MX, Zhuo K, Liao JL (2011) Multiplex PCR for the simultaneous identification and detection of *Meloidogyne incognita*, *M*. *enterolobii*, and *M*. *javanica* using DNA extracted directly from individual galls. Phytopathology 101: 1270–1277. doi: 10.1094/PHYTO-04-11-0095 21770774 10.1094/PHYTO-04-11-0095

[35] De Ley IT, De Ley P, Vierstraete A, Karssen G, Moens M, Vanfleteren J (2002) Phylogenetic analyses of *Meloidogyne* small subunit rDNA. J Nematol 34: 319–327. 19265950 PMC2620593

[36] Tigano MS, Carneiro RMDG, Jeyaprakash A, Dickson DW, Adams BJ (2005) Phylogeny of *Meloidogyne* spp. based on 18S rDNA and the intergenic region of mitochondrial DNA sequences. Nematology 7: 851–862.

[37] Humphreys-Pereira DA, Flores-Chaves L, Gomez M, Salazar L, Gomez-Alpizar L, Elling AA (2014) Meloidogyne lopezi n. sp. (Nematoda: Meloidogynidae), a new root-knot nematode associated with coffee (Coffea arabica L.) in Costa Rica, its diagnosis and phylogenetic relationship with other coffee-parasitising Meloidogyne species. Nematology 16: 643–661.

[38] Adams BJ, Dillman AR, Finlinson C (2009) Molecular taxonomy and phylogeny In Root-knot Nematodes, Eds Perry, Moens, and Starr. Publisher: CABI Publishing: 119–138.

[39] Carneiro RMDG, Correa VR, Almeida MRA, Gomes ACMM, Deimi AM, Castagnone-Sereno P, et al (2014) *Meloidogyne luci* n. sp. (Nematoda: Meloidogynidae), a root-knot nematode parasitising different crops in Brazil, Chile and Iran. Nematology 16: 289–301.

[40] Blok VC, Phillips MS, Fargette M (1997) Comparison of sequences from the ribosomal DNA intergenic region of *Meloidogyne mayaguensis* and other major tropical root-knot nematodes. J Nematol 29: 16–22. 19274129 PMC2619761

[41] Jepson SB, Golden AM (1987) *Meloidogyne marylandi* n. sp. (Nematoda: Meloidogynidae), a root-knot nematode parasitizing grasses Pp. 263–265 in Jepson S. B.. Identification of root-knot nematodes (*Meloidogyne* species). CAB International, Wallingford, United Kingdom.

[42] Starr JL, Ong KL, Huddleston M, Handoo ZA (2007) Control of *Meloidogyne marylandi* on bermudagrass. Nematropica 37: 43–49.

[43] Sekora NS, Crow WT, Mekete T (2012) First report of *Meloidogyne marylandi* infecting bermudagrass in Florida. Plant Dis 96: 1583–1584. 10.1094/PDIS-06-12-0544-PDN 30727335

[44] Walker N (2014) First report of *Meloidogyne marylandi* infecting bermudagrass in Oklahoma. Plant Dis 98: 1286. 10.1094/PDIS-04-14-0399-PDN 30699664

[45] Araki M (1992) The first record of Meloidogyne marylandi Jepson and Golden, 1987 from Zoysia spp. in Japan. Jap J Nematol 22: 49–52 (In Japanese).

[46] Oka Y, Karssen G, Mor M (2003) Identification, host range and infection process of Meloidogyne marylandi from turf grass in Israel. Nematology 5: 727–734.

[47] Salazar L, Gómez M, Flores L (2013) First report of *Meloidogyne marylandi* infecting bermudagrass in Costa Rica. Plant Dis 97: 1005. 10.1094/PDIS-11-12-1079-PDN 30722552

[48] Golden AM (1989) Further details and SEM observations on Meloidogyne marylandi (Nematoda: Meloidogynidae). J Nematol 21: 453–461. 19287638 PMC2618973

[49] Adam MAM, Phillips MS, Blok VC (2007) Molecular diagnostic key for identification of single juveniles of seven common and economically important species of root-knot nematode (Meloidogyne spp.). Plant Pathol 56: 190–197.

